# Selection of Intracellular Single-Domain Antibodies Targeting the HIV-1 Vpr Protein by Cytoplasmic Yeast Two-Hybrid System

**DOI:** 10.1371/journal.pone.0113729

**Published:** 2014-12-01

**Authors:** Julie Matz, Cécile Hérate, Jérôme Bouchet, Nelson Dusetti, Odile Gayet, Daniel Baty, Serge Benichou, Patrick Chames

**Affiliations:** 1 Inserm U1068, CRCM, Marseille, France; 2 Institut Paoli-Calmettes, Marseille, France; 3 Aix-Marseille Université UM105, Marseille, France; 4 CNRS, UMR7258, CRCM, Marseille, France; 5 Institut Cochin, CNRS UMR8104, Paris, France; 6 Université Paris Descartes, Paris, France; 7 Inserm U1016, Paris, France; Naval Research Laboratory, United States of America

## Abstract

The targeting of HIV-1 using antibodies is of high interest as molecular tools to better understand the biology of the virus or as a first step toward the design of new inhibitors targeting critical viral intracellular proteins. Small and highly stable llama-derived single-domain antibodies can often be functionally expressed as intracellular antibodies in the cytoplasm of eukaryotic cells. Using a selection method based on the Sos Recruitment System, a cytoplasmic yeast two-hybrid approach, we have isolated single-domain antibodies able to bind HIV-1 Vpr and Capside proteins in the yeast cytoplasm. One anti-Vpr single domain antibody was able to bind the HIV-1 regulatory Vpr protein in the cytoplasm of eukaryotic cells, leading to its delocalization from the nucleus to the cytoplasm. To our knowledge, this is the first description of a functional single-domain intrabody targeting HIV-1 Vpr, isolated using an *in vivo* cytoplasmic selection method that alleviates some limitations of the conventional yeast two-hybrid system.

## Introduction

Most antibody-based approaches to inhibit HIV-1 replication aim at neutralizing HIV-1 entry by targeting the Env protein [1], but many other HIV-1 proteins such as reverse transcriptase, integrase and protease enzymes, are efficient therapeutic targets, as demonstrated by their successful targeting by small inhibitor molecules used in infected patients in highly active antiretroviral therapy (HAART) [2]. These small molecules can efficiently bind the ligand binding site of their target, thereby inhibiting their function. However, while introduction of HAART have largely increased the survival time of HIV-1-infected patients, these therapies are unable to accomplish virus eradication in infected patients, indicating that targeting of other original viral determinants directly involved in HIV infection pathogenesis may have high beneficial impact, if combined with the current HAART regimens [3]. Protein-protein interactions represent major potential drug targets but these are unanimously difficult to consider with small chemical molecules. On the other hand, antibodies (Abs) are intrinsically endowed with the ability to interfere with a given protein-protein interaction [4], [5]. Unfortunately, most conventional Abs or their fragments, such as single-chain Fv fragments (scFvs), are not suitable for intracellular expression because their correct folding and stability generally depend on the formation of an intradomain disulfide bond, which cannot be efficiently formed in the reducing environment of the cytoplasm. Indeed, it has been shown that the stability of intrabodies is directly correlated to their performance when used as cytoplasmic inhibitors [6]. Few studies could isolate sufficiently stable scFv to demonstrate the feasibility of this approach to target HIV-1 proteins using anti-Tat or anti-Matrix scFvs [7], [8]. Single-domain antibodies (sdAbs), derived from heavy-chain immunoglobulins of Camelidae, are small (13 kDa) and highly stable antibody fragments that bind their target with high specificity and affinity in the nanomolar range [9]–[11]. Most of them can be functionally expressed into the cytoplasm [12]–[17] suggesting that disulfide bond formation is often not necessary to maintain their activity. As such they represent a rich source of functional intrabodies. Recently, we, and others, have used this property to isolate intrabodies against HIV-1 Nef and Rev proteins [18], [19] inhibiting most of the functions of these viral proteins.

One way to favor the efficient selection of functional intrabodies would be to perform their selection in an environment mimicking the cytoplasm of eukaryotic cells, unlike conventional methods such as phage display or ribosome display performed *in vitro*. To this aim, approaches based on yeast two-hybrid technology (Y2H) might be of special interest [20]–[22]. The conventional Y2H is based on a transcriptional readout where interaction between the bait and prey occurs in the yeast nucleus. Another advantage of *in vivo* selection methods such as Y2H is the absence of requirement for purified antigen, which can be tedious and time consuming to produce for *in vitro* selection approaches. However, despite its successfully use in many studies, conventional Y2H does suffer from some limitations. Indeed, interactions that involve transcriptional activators or repressors cannot be performed, and some proteins are toxic to yeast when targeted to the nucleus [23]. More generally, certain proteins may function more physiologically when expressed in the cytoplasm rather than in the nuclear milieu. To overcome these limitations, an alternative approach, named Sos Recruitment System (SRS), has been developed. SRS is a particular Y2H in which the interaction between bait and prey happens into the cytoplasm [24], alleviating several shortcomings of the conventional Y2H.

In this study, we provide a proof of concept of the feasibility to use SRS to isolate functional intrabodies targeting HIV-1 viral protein R (Vpr) and HIV-1 capsid (CA). Vpr is a viral accessory protein which disturbs many cellular pathways by interacting with cellular and viral proteins. Vpr is critical for efficient virus replication in macrophages, which are known to participate in virus dissemination and establishment of persistent virus reservoirs in different host tissues [25], [26]. CA is a part of the Gag polyprotein precursor and is crucial for the correct assembly of an infectious virion. CA is also involved in the decapsidation step, the reverse transcription and should also be involved, as well as Vpr, in the intracytoplasmic transport and nucleus import of the viral DNA [27], [28].

## Materials and Methods

### Immunization and phage display library construction

Two llamas were immunized with either a synthetic Vpr peptide (MEQAPEDQGPQREPYNDWTLELLEELKNEAVRHFPRIWLHSLGQHIYETYGDTWTGVEALIRILQQLLFIHFRIGCRHSRIGIIQQRRTRNGASKS) or recombinant CA produced in *E. coli* and purified via its GST Tag which was eliminated by thrombin cleavage. Using total RNA purified from llama PBMCs, single-domain antibody genes were amplified by RT-PCR and cloned into the pHEN1 phagemid as described [29], [30].

### Subcloning into pMyr vector

pMyr vector (CytoTrap Two-Hybrid System, Agilent technology) was modified by QuikChange to add SfiI and NotI restriction sites using primer newmyrfor (AATTTGCGGCCCAGCCGGCCCTCGTGATCAAACGGGCGGCCGCCG) and newmyrrev (TCGACGGCGGCCGCCCGTTTGATCACGAGGGCCGGCTGGGCCGCA) following the manufacturer's instructions. The resulting pMyr Sfi-Not was checked by sequencing and pHEN1 libraries were then subcloned into this vector using these restriction sites, as described [29], yielding pMyr-sdAb libraries.

### Cloning of vpr and CA genes into pSos

Vpr sequence was amplified by PCR from the pLEX-vpr plasmid using primers sosforvpr (AGTAGGATCCCCATGGAACAAGCCCCAGAAG) and sosrevvpr (TAATTAACCGCGGCGGCCGCTAGGATCTACTGGCTC CATTTC). CA sequence was amplified by PCR from the pLEX p24 CA plasmid using primers sosforCA (AGTAGGATCCCCATGGTACATCAGGCCATATCACC) and sosrevCA (TAATTAACCGCGGCGGCCGCTAC ATTGCTTCAGCCAAAAC). The amplified fragments were subcloned into vector pSos by classical NcoI and NotI digestion and ligation. Clones were verified by sequencing.

### Yeast Two-hybrid system - Sos Recruitment System (CytoTrap)

pSos-CA and pSos-Vpr were used as bait to screen the corresponding pMyr-sdAb libraries. The GAL1 promoter in the pMyr vector is repressed when glucose is used as a carbon source and quickly induced in the presence of galactose. Co-transfections were performed into the temperature-sensitive mutant *Saccharomyces cerevisiae* strain cdc25Hα (Agilent technology) using the standard lithium acetate method [31]. Negative controls were performed by using pSos-Vpr or pSos-CA and empty pMyr or irrelevant pMyr-Lam or pMyr-MAF-B vectors, and positive control was performed by using pMyr-Sos Binding protein (pMyr-SB) (Agilent technology). Resulting transformants were grown for 3 days at permissive temperature (24°C) onto selection medium containing glucose and additional supplements excluding leucine and uracil (SC-Leu–Ura) to select co-transfected yeast colonies. After replica-plating onto selective minimal galactose plates, colonies that showed growth under restrictive temperature (37°C) were considered positive clones (selection step). Next, selected colonies were picked on glucose containing medium and grown at 24°C for 2 days (amplification step). They were then replica-plated onto glucose or galactose plates and grew at 37°C (primary screening). To exclude false positives due to revertants, colonies which grew only onto galactose (inducing media) and not onto glucose plates were picked. This result was confirmed by a second round of screening performed on positive colonies by diluting them into 50 µl of sterile H_2_O and spotting 2 µl of each suspension onto glucose and galactose plates and incubated at 37°C (drop assay corresponding to a secondary screening). Colonies which grew onto galactose but not onto glucose plates were chosen as positive clones. DNA extraction of positive clones were done as described [31]. The extracted vector was used to transform bacteria for further amplification and sequenced.

### Drop Assay confirmation after fresh transformation

To further confirm the activity of each sequenced clone, a new drop assay was carried out using freshly transformed yeast. pMyr vectors encoding sequenced positive clones was used to co-transform the cdc25Hα yeast strain with the appropriate pSos vector. For each transformation, 3 colonies were diluted into 50 µl of sterile H_2_O and 2 µl of each suspension was spotted onto glucose and galactose plates and incubated at 37°C. Controls were performed using pSos-Nef, pSos-Vpr and pSos-CA with pMyr-V_H_H19 (V_H_H19 is an anti-Nef sdAb described previously [18]), pMyr-sdAb aCA2 (selected by phage display), an empty pMyr vector and pMyr-SB.

### Subcloning into pET vector for cytoplasmic expression

For cytoplasmic expression in *E. coli*, sdAb genes were subcloned into the pET-AviHis plasmid by using the InFusion kit (Clontech). Briefly, pET-AviHis was digested by NotI and NcoI enzymes, genes were amplified with pHENpETaviRev (CGTTCAGACCTGCGGCCGCTGAGGAGACAGTGACCTGG) and pHENpETaviFor primers (CTTTAAGAAGGAGATATACCATGGCCGAGGTGCAGCTGGTG) and subclonings were performed by recombination, in accordance with the manufacturer's indication. Clones were verified by sequencing.

### sdAb production and purification

*E. coli* BL21DE3 was transformed with pET vectors. Isolated colonies were used to inoculate 3 ml of 2YT broth containing 100 µg/ml Amp and 2% glucose that were incubated for 4 h at 37°C with agitation. These precultures were poured into 100 ml of 2YT containing 100 µg/ml Amp and shaked at 37°C until OD_600nm_ of 0.5 was reached. Then, 0.1 mM IPTG were added and temperature was decreased to 30°C and shaked overnight. Bacteria were pelleted and lyzed using BugBuster (Novagen) with lysozyme and benzonase. After centrifugation, the soluble fraction was purified by affinity on Talon beads (Clontech). Buffer was changed for PBS and proteins were concentrated using Vivaspin column (GE Healthcare). Concentration determination was done using Bradford protein assay (BioRad kit).

### ELISA assay

Maxisorp plates were coated with 10 µg/ml of GST-CA, GST-MA or Vpr at 4°C, overnight. Plates were saturated with 2% milk-PBS for 1 h. sdAbs Vpr1A and Vpr2A were added at 50 and 5 µg/ml and sdAbs CA7A and CA8B were added at 90 or 9 µg/ml for 1 h. sdAb aCA2 was used as positive control (50 µg/ml) and sdAb was omitted as negative control. After three 0.1% Tween-PBS (T-PBS) washes and three PBS washes, mouse anti-His6 antibody was added for 1 h. After another T-PBS and PBS washing, HRP-coupled anti-mouse antibody was added for 1 h. Revelation was done using ABTS. ODs at 450 nm were measured using a Tecan device.

### Subcloning into cell expression vector

SdAb genes and sequences coding for c-myc and His6 tags were amplified by PCR and subcloned into *XhoI* sites of pcDNA3.1 vector (Invitrogen). Plasmid encoding green fluorescent proteins (GFP) and hemaglutinin (HA)-tagged HIV-1 Vpr_LAI_ have been described previously [32].

### Cell culture

Hela-CD4 cell lines (TZM cells) were grown in Dulbecco's modified Eagle's medium (DMEM) supplemented with 10% fetal bovine serum and antibiotics at 37°C in a humid atmosphere containing 5% CO_2_.

### ImmunoFluorescence (IF)

250,000 HeLa cells were plated in 6-well plates containing 2 coverslips and were co-transfected with Fugene (Roche) the day after with 2 µg of plasmids coding either Vpr-GFP or Gag-GFP and 2 µg of the vector coding for one of the different sdAbs as recommended by the manufacturer. 24 h after transfection, cells were fixed with 4% paraformaldehyde for 20 min on ice. Cells were washed 3 times with PBS/BSA and stained with mouse anti-c-myc antibody 9E10 (1/2000, Roche) in PBS/BSA supplemented with Triton X-100, 0.1% (Sigma-Aldrich) during 1 hour on ice. After another PBS/BSA washing, 1/250 dilution of goat anti-mouse conjugated-AlexaFluor555 (Molecular probes, Invitrogen) was added for 1 hour on ice and washed again with PBS. Cells were plated on microscope slides in a PBS-glycerol mix (50∶50) using the SlowFade Light Antifade Kit containing DAPI (Molecular probes, Invitrogen). Cells were imaged using a spinning disk confocal imaging system (CSU-X1M1, Yokogawa) based on an inverted microscope (Leica DMI6000) with a CoolSnap HQ^2^ camera (Photometrics) with a 100× HCX PL APO objective. The acquisition of images was done with MetaMorph 7.5 (Molecular Devices). The coefficient of Pearson which illustrates the co-localization ratio has been calculated using the Fiji software.

To confirm results with sdAb Vpr1A, the same experiment was done with different ratios (1∶1, 2∶1 and 4∶1) of sdAb Vpr1A and HA-Vpr vectors with additional steps of antibody incubations with the rat anti-HA 3F10 monoclonal antibody (1/300, Roche) for 1 hour followed by goat anti-rat conjugated-AlexaFluor488 (1/250, Molecular probes, Invitrogen) for 1 h.

To further study sdAbs CA7A and CA8B, the same experiment was performed with a HA-CA construction. Cells were treated in the same way as the ones transfected with HA-Vpr. Plasmid coding for HA-CA was a kind gift of Dr E. Le Rouzic (Cochin Institute, Paris, France).

### Cell cycle analysis

5×10^6^ HeLa cells were transfected by electroporation (200V/950µF) with 5 µg of the GFP expression plasmid, 8 µg of the plasmid encoding HA-Vpr and different amounts of the sdAb-Vpr1A plasmid. 2×10^6^ transfected cells were plated for cell cycle analysis while 3×10^6^ cells were plated for immunoblotting analysis. Two days later, cells were fixed for 15 min in 1% PFA, permeabilized in cold ethanol for 1 h at 4°C, resuspended in PBS containing 200 µg/ml RNase and 50 µg/ml propidium iodide (PI), and incubated for 15 min at room temperature prior to analysis of DNA content by flow cytometry as described previously [33], on a minimum of 5,000 GFP-positive cells using a Cytomics FC 500 instrument (Beckman Coulter). The G2 arrest activity was evaluated using the FlowJo software and we quantified the cells in the different cell cycle phases using the Dean-Jett-Fox model [34].

### Apoptosis assay

15×10^5^ HeLa cells were plated in 6-wells plates and transfected with 1.3 µg of GFP expression plasmid, 2 µg of the plasmid encoding HA-Vpr and different amounts of the plasmid encoding sdAb Vpr1A. The apoptosis assay was performed three days after transfection, cells were assayed for cell surface exposure of phosphatidylserines (PS) using the PE AnnexinV Apoptosis Detection kit 1 from BD Pharmingen, as recommended by the manufacturer, and analyzed by flow cytometry on a minimum of 5,000 GFP-positive cells using a Cytomics FC 500 instrument (Beckman Coulter).

## Results

Our goal was to isolate llama derived single-domain antibodies (sdAbs) against HIV-1 Vpr and CA proteins. Our first attempt was to immunize llamas with recombinant antigens and to isolate sdAbs using a well established phage display procedure [29], [30] on plastic or bead-immobilized antigens. Two llamas were immunized with a 96 amino acid-long synthetic polypeptide corresponding to the full length Vpr proteins or a recombinant CA protein produced in *E. coli*. The corresponding phage display libraries built using sdAb genes amplified by RT-PCR from llama PBMCs and based on pHEN1 phagemid vector had a diversity of 5×10^7^ and 1.3×10^7^ clones for Vpr and CA, respectively. Classical phage display enrichment procedures performed on synthetic Vpr peptide adsorbed on plastic or covalently bound to magnetic beads did not lead to specific binders. Similar selection procedures performed on recombinant CA were successful, and the sequencing of positive binders revealed a unique clone, called sdAb aCA2 (unpublished results).

**Figure 1:**
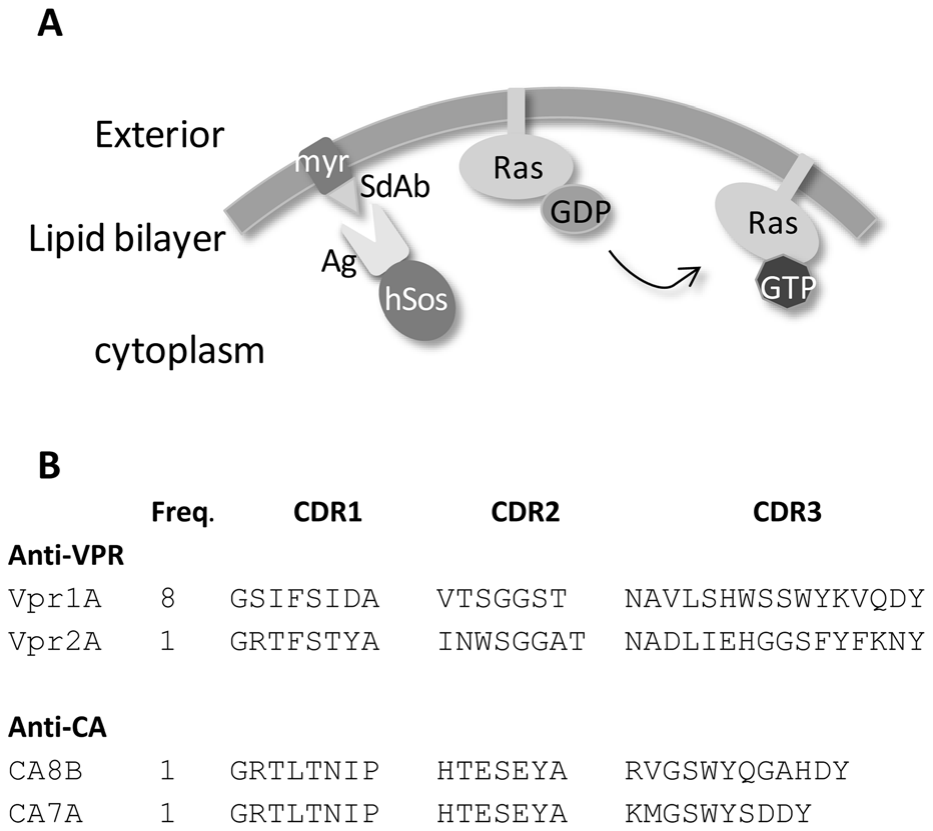
Selection system principle and sequences of selected binders. **A)** *Representation of the Sos Recruitment System principle*. The antigen (Ag) is fused to the human Sos protein (hSos) while sdAbs are fused to a myristoylation signal anchoring them in the plasma membrane. If the sdAb binds its Ag, hSos is driven to the membrane and activates the Ras pathway, leading to the growth of cdc25H mutant yeast at non-permissive temperature. **B)** *Sequences of clones isolated by the SRS technique*. Two anti-Vpr and 2 anti-CA sdAbs were isolated by SRS. The sequences of their 3 complementary determining regions (CDRs) are shown. The frequency of each clone in the selection output (Freq.) is also reported.

Consequently, we decided to compare the selection efficiency using this time a cytoplasmic yeast two-hybrid approach named *Sos Recruitment System* (**Fig. 1A**). This approach does not rely on the integrity of a purified antigen and circumvents possible conformation issues of immobilized antigens during phage display selections. The two llama sdAb libraries were thus subcloned into pMyr vector (CytoTrap SRS system), yielding 9×10^7^ and 3.3×10^7^ colonies, thereby preserving most of the initial library diversities.

### In vivo selection in yeast by Sos Recruitment System

Large preparation of the corresponding pMyr vector libraries were performed and used for yeast co-transformation experiments with pSos vectors encoding the fusion of human Sos to the corresponding Vpr or CA antigen as baits. 10^7^ and 1.4×10^6^ yeast colonies clones were obtained using Vpr or CA, respectively, after incubation at 24°C (**Table 1**). The sizes of these new libraries were found satisfactory as they correspond to immunized animals.

**Table 1:** Number of clones at each step of the selection process.

	Diversity of bacteria libraries	Number of yeast transformants	Number of selected clones: selection/primary screening/secondary screening	Number of clones after sequencing
Vpr	9×10^7^	10^7^	850/13/11	2
CA	3.3×10^7^	1.4×10^6^	475/2/2	2

The selection round yielded 850 and 475 positive clones for Vpr and CA, respectively. A screening step based on the same interaction (see materials and methods for details) decreased the number of positive clones to 13 and 2 positives clones for Vpr and CA, respectively (**Table 1**). During a second screening step based on a more stringent drop assay, two anti-Vpr colonies were found to be false positives, decreasing the number of candidate to 11 for Vpr and 2 for CA. The pMyr vectors of these clones were purified and sequenced. The anti-Vpr clones yielded two unrelated sequences named Vpr1A and Vpr2A. The two anti-CA clones, named CA7A and CA8B, share their CDR1 and CDR2, but show some differences in the sequence and length of their CDR3 (**Fig. 1B**).

### Drop Assay after fresh transformation

To confirm these interactions and further establish the specificity of these sdAbs, each clone was submitted to a new drop assay based on freshly transformed yeasts, using Vpr, CA or an empty pSos vector as negative control. The interaction between Nef and an anti-Nef sdAb (V_H_H19) was used as positive control in this assay. A pMyr vector coding for a Sos binding protein, able to interact with any hSos fusion was used to ensure the expression of each fusion protein (**Table 2**). As expected, co-transformations of pMyr-Vpr1A and pMyr-Vpr2A with pSos-Vpr led to yeast colonies onto galactose medium but not onto glucose plate at non-permissive 37°C temperature, as did co-transformation of pMyr-CA7A and pMyr-CA8B with pSos-CA, as well as pMyr-V_H_H19 and pSos-Nef. All pSos plasmids led to colonies when co-transformed with pMyr-SB. No other interaction was detected on galactose plate, establishing the specificity of these interactions. Interestingly sdAb aCA2, selected by phage display, was not able to interact with hSos-CA in this setting.

**Table 2:** Drop assay after fresh transformation.

pMyr	SB	VHH19	aCA2	CA7A	CA8B	Vpr1A	Vpr2A	Empty
pSos	+++	-	-	-	-	-	-	-
pSos-Nef	+++	+++	-	-	-	-	-	-
pSos-Vpr	+++	-	-	-	-	+++	+	-
pSos-CA	+++	-	-	+	+	-	-	-

Yeast co-transfected with either pSos-Nef, pSos-Vpr or pSos-CA or pSos (empty vector) and either pMyr-VHH19 (an anti-Nef sdAb previously described), pMyr-aCA2 (selected by phage display), pMyr (empty), pMyr-SB (Sos binding protein), or pMyr plasmids coding for Vpr1A, Vpr2A, CA7A or CA8B were resuspended and spotted onto galactose or glucose plates. Growth of colonies on galactose plates was indicated by + or - according with number and size of colonies.

### Purification from the cytoplasm of *E. coli*

**Figure 2:**
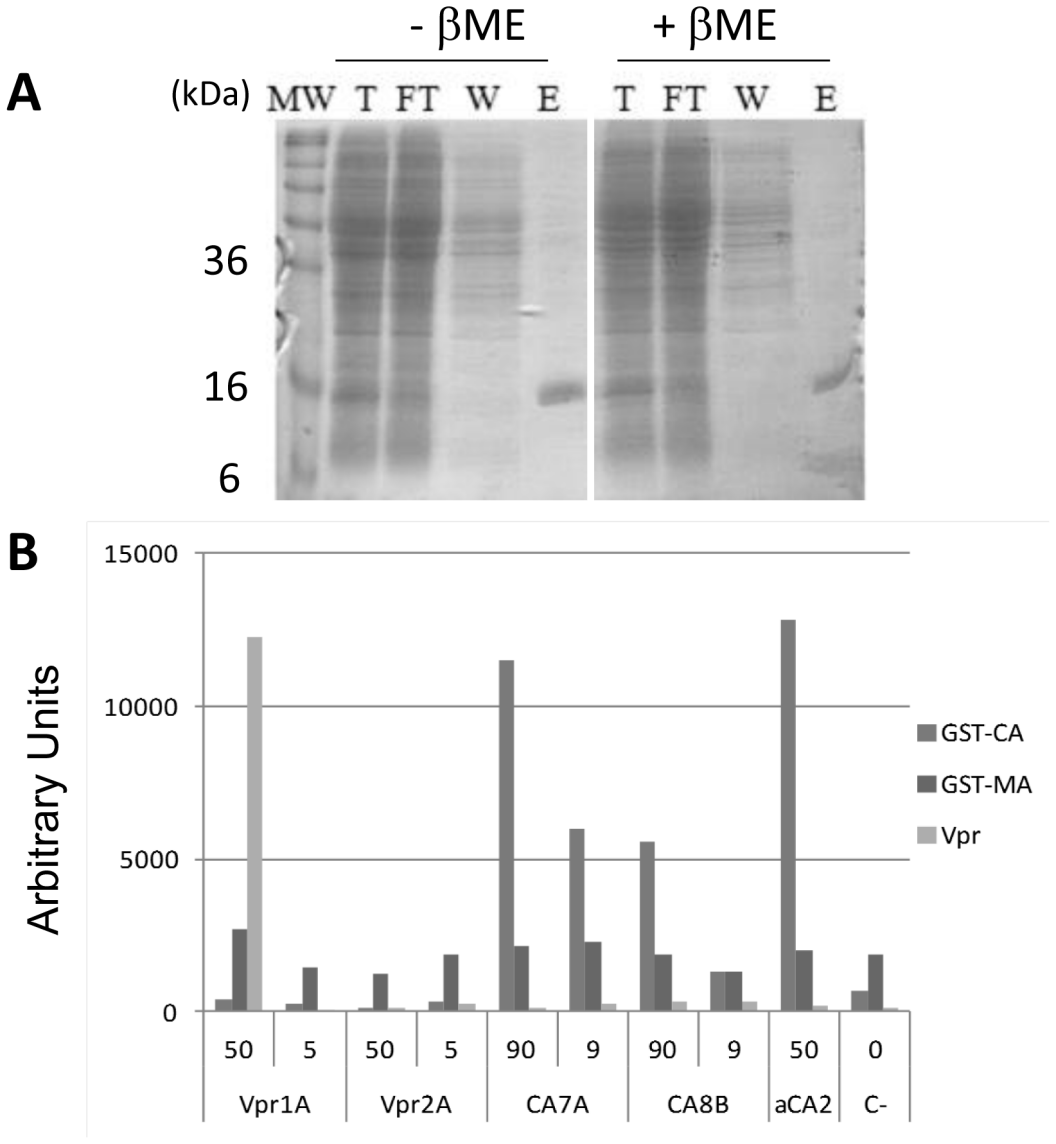
sdAb biochemical characterization. **A**) *Purification of sdAbs produced into the cytoplasm of E. coli*. SDS-PAGE gel analysis was performed using comparable amount of sample for each step of the hexahistidine tag based purification process. Molecular weights (MW) are indicated. Soluble fraction of the total lysate (T), flow through (FT), washes (W) and elution fraction (E) were loaded in the presence or absence of β-mercaptoethanol (βME). Depicted results corresponding to sdAb Vpr1A are representative of what was obtained for other clones. **B**) *ELISA assay* w*ith purified sdAbs*. ELISA was performed by coating Maxisorp plates ON at 4°C with 10 µg/ml of GST-CA, GST-MA or Vpr. sdAbs were added at indicated concentrations (µg/mL). sdAb aCA2 was used as positive control (50 µg/ml) and the absence of sdAb was used as negative control.

Such sdAbs capable of functional binding in the cytoplasm of yeast should also be produced in a soluble form in the cytoplasm of *E. coli*, where overexpressed but unfolded proteins usually form insoluble inclusion bodies. The four sdAbs were thus produced in the cytoplasm of *E. coli* and purified from the soluble fraction using metal affinity chromatography. All sdAbs could be easily produced and purified, reaching production yields of 38, 32, 35 and 30 mg/L of culture for Vpr1A, Vpr2A, CA7A and CA8B, respectively. **Fig. 2A** shows the analysis of the different fractions for sdAb Vpr1A. Interestingly, no difference of migration was observed in the presence or absence of β-MercaptoEthanol (βME), suggesting that no disulfide bond was formed. Overall, these successful purifications indicate that these sdAbs can remain soluble when expressed at high yield in the reducing environment of the *E. coli* cytoplasm. The purified sdAbs were assayed for binding to recombinant GST-CA, GST-MA and synthetic Vpr by ELISA, despite some concerns about possible changes of antigen confirmation induced by direct adsorption on plastic, that could be detrimental to the binding of conformation-sensitive binders. sdAb Vpr1A was found positive and specific for Vpr, confirming the Y2H result, whereas Vpr2A was found negative in this setting (**Fig. 2B**). sdAbs CA7A and CA8B were also found positive and specific for CA in this assay.

### Colocalization experiments by immunofluorescence

**Figure 3:**
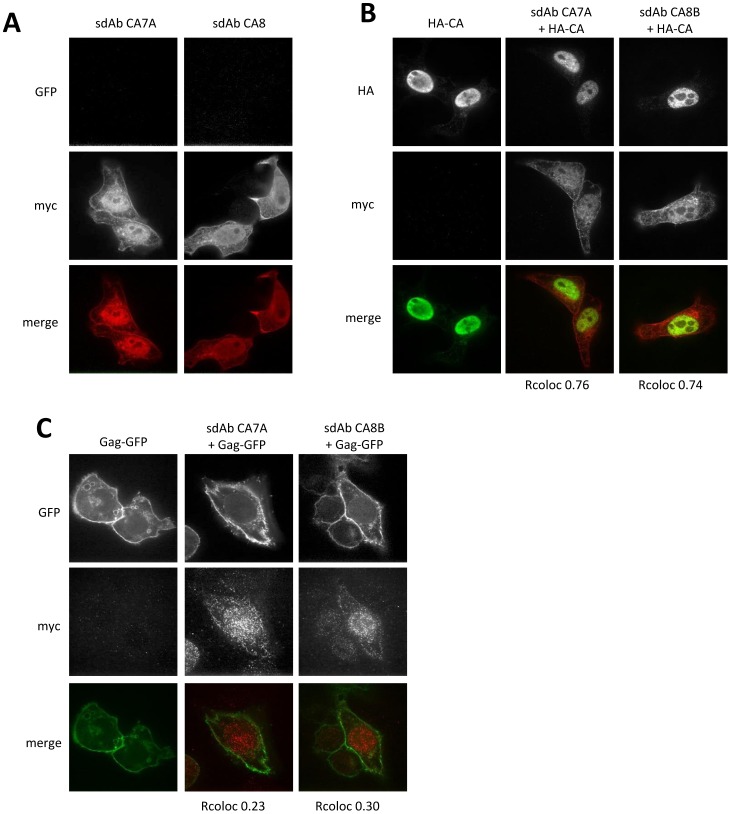
Immunofluorescence assay using Gag-GFP, HA-CA and sdAbs CA7A and CA8B into HeLa cell. Immunofluorescence was performed by co-transfecting HeLa cells with a plasmid bearing HA-CA gene (**B**) or Gag-GFP gene (**C**) and a plasmid bearing a sdAb gene as indicated. Gag-GFP was tracked by GFP fluorescence. HA-CA was tracked using an anti-HA rat monoclonal antibody followed by an anti-rat monoclonal antibody coupled to AlexaFluor488 (green). SdAbs were detected using a mouse anti-c-myc monoclonal antibody followed by an anti-mouse antibody coupled to AlexaFluor555 (red). **Fig. 3A** are control cells transfected with the sdAb alone. **Fig. 3B** show cells transfected with HA-CA and **Fig. 3C** cells transfected with Gag-GFP. The coefficient of Pearson (coefficient of co-localization) was calculated for each co-transfected cell.

To be used as intrabodies, these sdAbs must be able to bind their target in eukaryotic cells. To evaluate this possibility, the genes coding for these sdAbs fused to a c-myc epitope at their C-terminus were subcloned into pcDNA3 and co-transfected together with HA-tagged or GFP fusions of the viral targets. The sdAb expression and cellular localization was followed using an anti-c-myc tag monoclonal antibody. sdAbs CA7A and CA8B were properly expressed and equally distributed between cytoplasm and nucleus (**Fig. 3A**). When co-expressed with HA-CA, which mainly localized in the nucleus, sdAbs CA7A and CA8B showed a significant co-localization with CA in the nuclear compartment (**Fig. 3B**), as indicated by calculation of the Pearson's coefficient (*r* = 0.76 and 0.74, respectively). However, they poorly co-localized with Gag-GFP which was mainly localized at the cell periphery (**Fig. 3C**, *r* = 0.23 and 0.30).

**Figure 4:**
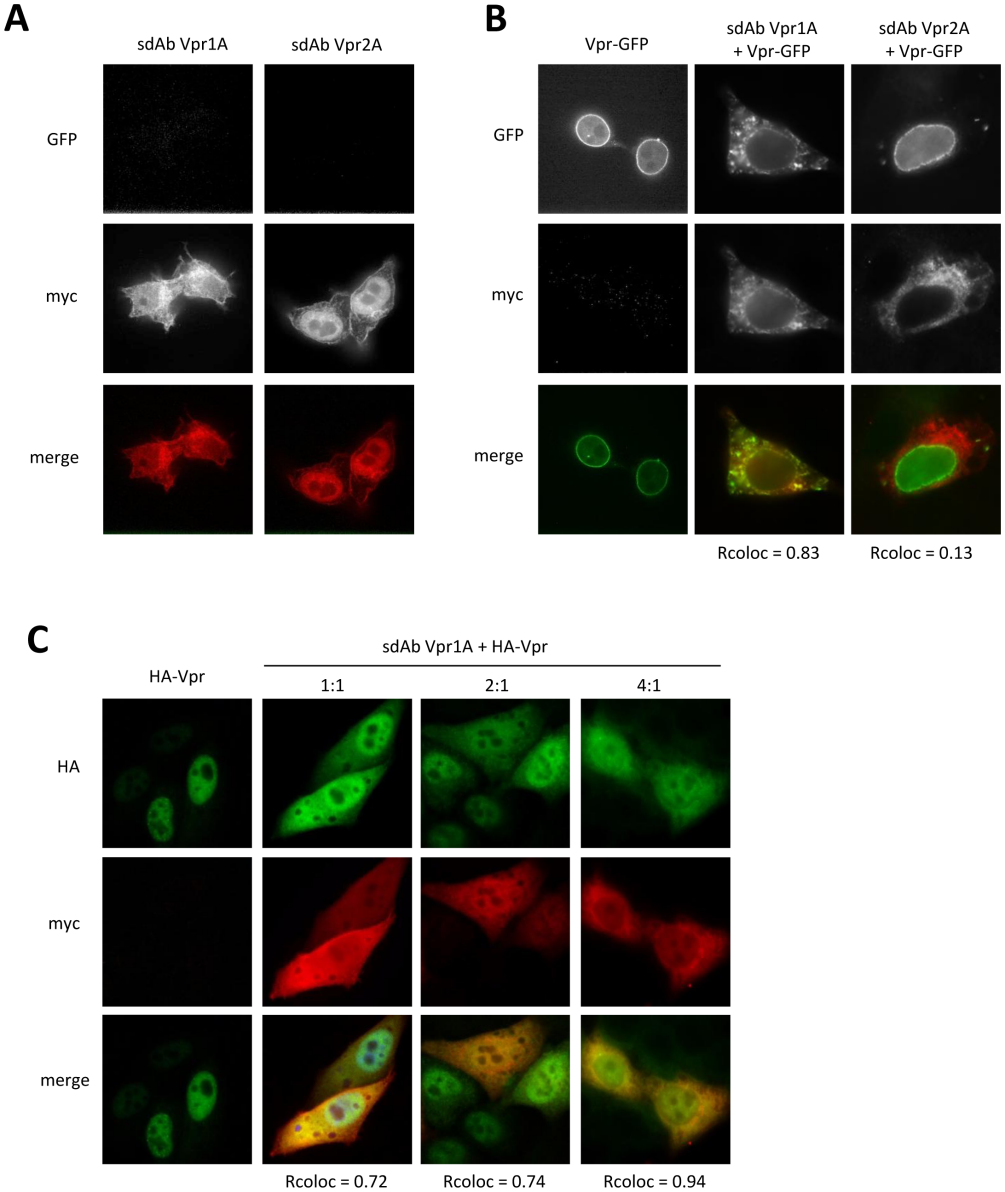
Immunofluorescence assay using Vpr-GFP, HA-Vpr and sdAbs Vpr1A and Vpr2A. Immunofluorescence was performed by cotransfecting Hela cells with a plasmid coding for Vpr-GFP (**B**) or HA-Vpr (**C**) and increasing amount of a plasmid coding for a sdAb. Vpr-GFP, HA-Vpr and sdAbs were tracked as described in **Fig. 3**. The coefficient of Pearson (coefficient of co-localization) was calculated for each cell cotransfected with Vpr-GFP and a sdAb.

Similarly anti-Vpr sdAbs were properly expressed and were distributed between the cytoplasm and the nucleus of cells (**Fig. 4A**). In the absence of sdAbs, Vpr-GFP was localized in the nucleus and also concentrated at the nuclear envelope (see **Fig. 4B**). The presence of sdAb Vpr2A did not have any effect on the nuclear localization of Vpr-GPF (**Fig. 4B**, right column). Indeed, the sdAb was not detected in the nucleus, indicating that this clone was not able to bind Vpr in this setting. This result is in agreement with *in vitro* ELISA results shown in Fig. 2B. Conversely, the presence of sdAb Vpr1A led to a major delocalization of Vpr-GFP in the cytoplasm compartment (**Fig. 4B**, middle column). Vpr-GFP and sdAb Vpr1A showed a high degree of co-localization, as illustrated by the Pearson's coefficient (*r* = 0.83), implying that the sdAb was able to sequestrate a very significant part of Vpr-GFP in the cytoplasm, thereby severely reducing its concentration in the nucleus.

To rule out any artifact that might be generated by the presence of GFP, and to define more precisely the effect of sdAb Vpr1A, a similar experiment was performed in cells co-expressing sdAb Vpr1A and Vpr fused to the small HA epitope tag (HA-Vpr) for detection (**Fig. 4C**). In the absence of sdAb Vpr1A, HA-Vpr was strictly localized in the nucleus (**Fig. 4C**, first column). When expressed in the presence of SdAb Vrp1A, HA-Vpr was found co-localized with the sdAb in the cytoplasm and in the nucleus, independently of the amount of sdAb coding plasmid used for transfection (**Fig. 4C**). This observation confirmed that anti-Vpr sdAb Vpr1A can efficiently bind its antigen in the eukaryotic cytoplasm environment. Moreover this interaction clearly interferes with Vpr cellular localization.

### Vpr-induced cell cycle arrest and apoptosis

**Figure 5:**
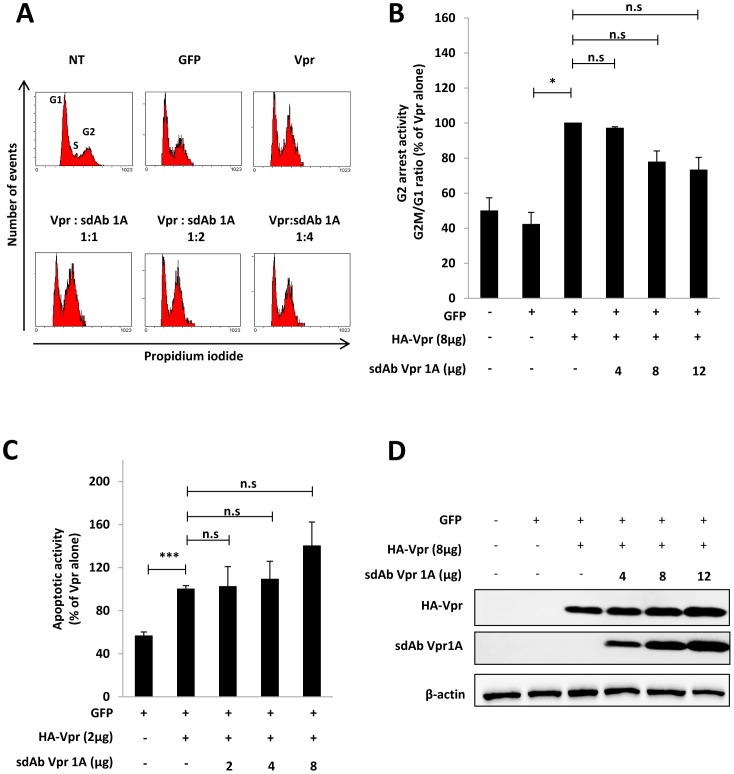
Impact of sdAb-Vpr1A on the G2-arrest and pro-apoptotic activities of Vpr. HeLa cells were co-transfected with plasmids for expression of GFP and HA-Vpr in combination with increasing concentrations of c-myc tagged sdAb Vpr1A expression plasmid when indicated. (**A, B**) *Cell cycle analysis*. 48 h after transfection, cells were fixed, permeabilized, and stained with propidium iodide. The DNA content was analyzed by flow cytometry on GFP-positive cells. In **A**, the cell DNA content profiles from a representative experiment are shown. The cells in G1, S and G2/M phases are indicated on the upper right panel. In **B**, results are expressed as the percentage of the G2M/G1 ratio relative to that measured in cells expressing HA-Vpr alone and are the means of 3 independent experiments. Error bars represent 1 S.D. from the mean. Statistical significance was determined using students *t* test (n.s., p>0.05; *, p<0.05). **C**) *Pro-apoptotic activity*. 72 h after transfection, cell surface PS exposure was analyzed by flow cytometry on GFP positive cells after staining with phycoerythrin-labelled Annexin V and 7AAD (7-Aminoactinomycin). Results are expressed as the percentage of GFP-positive cells displaying surface PS exposure  =  relative to cells expressing HA-Vpr alone, and are the means of 3 independent experiments. Error bars represent 1 S.D. from the mean. Statistical significance was determined using students *t* test (n.s., p>0.05; *, p<0.05; **, p<0.01;***, p<0.001). **D**) *Expression of Vpr and sdAb Vpr1A proteins*. Lysates from HeLa transfected cells were analyzed by Western blotting using anti-HA (upper panel), anti-c-myc (middle panel) and anti-β-actin antibodies.

Since the G2-arrest activity and apoptosis induction are the main documented functions of Vpr ([35] for review), we wanted to evaluate whether intracellular sdAb Vpr1A could interfere with the cytostatic and apoptotic effects mediated by Vpr. First, we studied the cytostatic activity of Vpr leading to an arrest of the cell cycle in the G2 phase. HeLa cells were co-transfected with plasmids encoding for HA-Vpr and c-myc-tagged sdAb-Vpr1A together with a GFP expression vector, and the DNA content was analyzed 48 h later by flow cytometry on GFP-positive cells after staining with propidium iodide. The DNA content profiles of transfected cells from a representative experiment is shown in **Fig. 5A**, and the results of 3 independent experiments are shown in **Fig. 5B**. In the absence of sdAb Vpr1A, efficient G2-arrest was measured in cells co-expressing HA-Vpr and GFP, as evidenced by the net increase of the G2M/G1 ratio compared to cells expressing GFP alone (**Fig. 5B**). However, when cells were co-transfected with increasing amount of the sdAb-Vpr1A expression plasmid, no apparent inhibition of the G2-arrest induced by HA-Vpr was observed, even though sdAb Vpr1A was correctly expressed in co-transfected cells (**Fig. 5D**).

The potential effect of sdAb Vpr1A on the Vpr proapoptic activity was then assayed, 72 h after transfection, by flow cytometry analysis of the cell surface exposure of phosphatidylserine (PS) after staining with phycoerythrinlabeled Annexin V (**Fig. 5C**). As expected, Vpr expression led to an increase of apoptotic cells but the co-expression of sdAb-Vpr1A had no inhibitory activity on the Vpr-induced apoptotic effect. Altogether, the results reported in Fig. 5 indicate that, despite its strong effect on Vpr localization, sdAb Vpr1A has no effect on these two main Vpr functions.

## Discussion

In this study, we used an alternative yeast two-hybrid system to select single-domain antibodies able to properly fold and bind their antigen in the yeast cytoplasm. The so-called Sos Recruitment System was used to isolate binders from two libraries of sdAbs built from the blood of two llamas immunized with either a recombinant HIV-1 capsid protein or a synthetic HIV-1 Vpr peptide. Two sdAbs could be selected for each target. An independent *in vitro* assay using purified sdAbs on purified antigens adsorbed on plastic confirmed the interaction for one sdAb for Vpr and two sdAbs for CA. Finally, immunofluorescence was used to demonstrate that anti-CA sdAbs could partly co-localize with their target in cells, while the Vpr binder was able to significantly alter the cellular localization of its antigen.

*In vitro* phage display selections on immobilized recombinant CA protein selected a single sdAb (aCA2). The same sdAb library subjected to the *in vivo* Y2H selection procedure yielded two other clones, CA7A and CA8B, that were not found in the phage selection output. Interestingly, aCA2 selected by phage display, i.e. for its ability to bind its antigen in regular conditions, was found negative when tested as intrabody in the Y2H assay. These results highlight the fact that a selection method should ideally be adapted to the final use intended for the selected binders. Unfortunately, none of the two capsid binders, interacting properly with CA in the yeast cytoplasm, was found able to interact with a GFP fusion of the Pr55Gag precursor. It cannot be ruled out that these sdAbs are simply not functional when expressed in the cytoplasm of eukaryotic cell. Another explanation might be that the epitope targeted by these sdAbs on the mature capsid is not accessible in the Pr55Gag polyprotein before the proteolytic maturation process. This second explanation is supported by the results obtained when sdAbs CA7A and CA8B were co-expressed with the HA-tagged form of CA, leading to a to significant co-localization of both sdAbs and HA-CA in the nucleus, thereby demonstrating a binding activity. Since the maturation step of Pr55Gag takes place in a late stage of the replication process [27], it would have been more relevant to directly select for Gag binders. Unfortunately, sufficient amounts of a recombinant and purified Pr55Gag polyprotein was not available for the immunization step.

Vpr is a small protein which is also difficult to produce recombinantly but which can be obtained by peptide synthesis. However, the structure of such a peptide might be different from the native peptide. In fact several conformations might be present in such sample. Phage display selection did not yield any binder, which might be related to this issue or to more trivial issues such as the adsorption efficiency of Vpr on solid surface during panning and screening steps. On the other hand, two binders were selected *in vivo* by Y2H, and one of these binders was positive by ELISA and co-localized with Vpr in human cells by immunofluorescence assay. Interestingly, a large proportion of Vpr-GFP or HA-tagged Vpr was sequestered in the cytoplasm when co-expressed with this sdAb. We and others have already observed such phenomenon using sdAb-based intrabodies [18], [19]. Using an anti-Nef intracellular sdAb, we could efficiently block most of the activities of this protein [18]. Interestingly, by fusing this sdAb to a SH3 domain targeting a different epitope of Nef via a flexible linker peptide, we could block all known activities of HIV-1 Nef [36], [37]. The mechanisms by which the sdAb induces Vpr delocalization deserve further studies. One possibility might that the sdAb targets the nuclear localization signal of Vpr, and thus, inhibits the nuclear import, as it has been shown for phage display-selected scFvs [38]. Alternatively, the inhibition might be due to steric hindrances generated by the large size of sdAb-Vpr complex. Surprisingly, this strong effect on Vpr localization did not translate into an inhibition of two Vpr main functions, i.e. cytostatic and pro-apoptotic effects. Possible explanations might be that the residual amount of Vpr remaining in the nucleus is able to induce these effects, and/or that the Vpr epitope bound by sdAb Vpr1A is not a Vpr epitope involved in these effects.

Other Y2H studies have selected single-chain antibodies [21], [39], single-chain intrabodies [40] or even single-domain antibodies [41], but always using nuclear Y2H. To our knowledge this work is the first to demonstrate that a cytoplasmic two-hybrid approach in yeast can be used to isolate antibody fragments targeting intracellular antigens, i.e. intrabodies. Interestingly, bacterial-two-hybrid selections of sdAb-based intrabodies has recently been reported [42]. However, the yeast approach brings the possibility of targeting antigens subjected to post-translational modifications, which increases the chance of isolating intrabodies recognizing their antigen in their native state in the mammalian cytoplasm.

By using a synthetic Vpr polypeptide to immunize llamas, we successfully isolated a single domain intrabody able to bind Vpr in the cytoplasm of eukaryotic cells. One drawback of this approach is the low number of sdAbs selected in this work. A larger number of positive clones could possibly be achieved by increasing the size of the original yeast library via larger scale transformation reaction. The quality of the library is also dependent on the immunization step that could be improved via the use of immunogenic carrier, especially for small proteins such as Vpr (13 kDa). Nevertheless, we obtained a sdAb able to interfere with Vpr cellular localization. To our knowledge, it is the first sdAb and the first intrabody targeting the HIV-1 Vpr protein. This sdAb should be useful as a tool for functional studies of Vpr and might also help in the design of Vpr inhibitors or even be used as *in vivo* tools for therapeutic applications. For example, an innovative bispecific reagent could be generated by fusing the available anti-Nef intrabody with the anti-Vpr intrabody generated in this study. Such a construct might be instrumental in the study of the virus replication and it would be of high interest to see if such a reagent can efficiently hinder the replication and infection efficiency of the virus.
